# The Individual as Moderator of Variable Relations – A Configural Approach

**DOI:** 10.17505/jpor.2024.26256

**Published:** 2024-05-23

**Authors:** Alexander von Eye, Wolfgang Wiedermann

**Affiliations:** 1 Michigan State University, USA; 2 University of Missouri, Columbia, USA

**Keywords:** moderator variables, moderator models, person-oriented research, configural frequency analysis, individuals as moderators

## Abstract

Moderators are variables that change the relations among other variables. Moderators are variables that are substantive just as the variables whose relations are moderated. In the present article, we propose using individuals as moderators. Specifically, we propose using Configural Frequency Analysis, that is, investigating moderators from a person-oriented perspective. The question asked is whether variable relations vary across individuals. Base models are specified for Configural Frequency Analysis that allow one to identify individuals that differ in variable relations. In a data example, it is shown that not a single individual in a sample of alcoholics shows the pattern of association between subjective stress and beer consumption that was found for the entire sample. Extensions of the configural moderator model are discussed.

## Introduction

*Moderators* are defined as variables that change relations among other variables as scores move along a scale (when they are continuous) or across categories (when they are categorical) (Baron & Kenny, 1986; von Eye & Wiedermann, 2023). To give an example with continuous variables, consider the effect that a medicinal drug has on headaches. This effect can vary depending on the amount of alcohol consumed by a patient. Similarly, in categorical variables, the effect of red wine consumption may depend on the gender of the consumer.

A number of methods have been proposed to assess moderator effects. In continuous variable contexts, moderator effects are often estimated by way of calculating the interaction between the moderator and the independent variable(s) (see e.g., Baron & Kenny, 1986; Park & Yi, 2022; von Eye & Wiedermann, 2023), so-called moderated regression models. Consider the regression model


$$y=\beta_{0}+\beta_{1}x+\varepsilon_{1}.$$

where

*y* is a value of the dependent variable *Y*,

*x* is a value of the independent variable *X*,

𝜖_1,_ is the error,

β_0_ is the intercept, and

β_1_ is the regression slope parameter.

Now, let the hypothesis be proposed that variable *Z* moderates the *X* – *Y* relation. Then, one approach to assessing the strength of the moderator effect is to include *Z* as well as the *Z* × *X* interaction in the regression model. The model thus becomes


$$y=\beta_{0}+\beta_{1}x+\beta_{2}z+\beta_{3}xz+\varepsilon_{2}.$$

Depending on the variable situation at hand, more elaborated models can be considered. This applies in particular when collinearity is present (Park, & Yi, 2022; von Eye, & Wieder-mann, 2023) or when nonlinear relations are investigated.

In the context of categorical variables, moderator effects can be assessed in an analogous fashion. It is one option to specify models parallel to the regression models above. Consider the log-linear regression model (cf. von Eye et al., 2005)


$$\log\hat{m}=\lambda_{0}+\lambda_{1}^{x}+\lambda_{2}^{y},$$

where

$\hat{m}$ is the estimated cell frequency,

𝜆_0_ is the model constant,

*y* is a category of the dependent variable Y,

*x* is a category of the independent variable X, and

𝜆_1_ and 𝜆_2_ are the corresponding effect parameters.

Accordingly, when *Z* is considered a moderator, the model becomes


$$\log\;\hat{m}=\lambda_{0}+\lambda_{1}^{x}+\lambda_{2}^{y}+\lambda_{3}^{z}+\lambda_{4}^{xz}.$$

It should be noticed that, when *Z* is included in the cross-classification under study, the table will be larger than without *Z*. Specifically, let the cross-classification without *Z* have *t* cells, and let *Z* have *j* categories. Then, the cross-classification for the model that includes *Z* will have *t* × *j* cells. In loglinear analysis, this will cause problems only when the sample is small (von Eye, & Mun, 2013 b). In configural analysis, however, this increase in the number of cells can cause problems because the protected significance threshold, α*, will be more extreme by a factor of *j*, and will, thus, become more prohibitive (see von Eye, & Wiedermann, 2021).

As an alternative to the approach that uses logit models of the entire cross-classification, one can consider estimating the simpler model separately for each of the categories of *Z*. The moderator effect can be considered established when the parameters of the separate models differ.

Clearly, the moderator models reviewed so far are person-oriented in the sense that they allow one to test whether parameters differ across a priori specified groups of individuals, that is, the individuals that exhibit the various categories of the moderator, *Z*.

In the present article, we propose three new approaches to moderator analysis. First, we go one step farther in the direction of person orientation. Instead of testing hypotheses in which variables are moderators, we look at individuals. We ask whether individuals can be moderators themselves. In other words, we ask whether the results of data analysis differ across individuals.

This question lies in the heart of person-oriented research. The first tenet of this research orientation states that parameters are person-specific (Bergman, & Magnusson, 1993; von Eye, & Bergman, 2003). When individuals are moderators themselves, parameters are estimated so that the ‘variable’ that indexes the individuals is the moderator or, parameters are estimated separately for each individuals and then, compared.

The second new element proposed in this article is that we do not estimate regression models, neither linear nor logit models. Instead, we propose using individuals as moderators in a configural analysis. Specifically, we propose performing configural frequency analysis (CFA; see Lienert, 1968; von Eye, & Wiedermann, 2021) such that the ‘variable’ that indexes the individuals is included in the model.

The third new element proposed in this article concerns the characteristics of the ‘moderators,’ that is, the individuals that differ in their variable relations. We ask whether individuals with similar variable relations can be discriminated based on variables that are not included in the original moderator analysis. Here, again, we propose configural models instead of models that relate variables to each other.

## CFA when the Individuals are the Moderator

In contrast to most other methods of statistical data analysis, CFA allows researchers to ask questions concerning individual cells or groups of cells in cross-classifications. These questions are specified in the form of *CFA base models*. These models contain all effects that are *not of interest* to the researcher. When such a model is rejected, the effects of interest are bound to exist. The researcher then identifies those sectors in the data space, that is, those cells or groups of cells that contradict the base model significantly. When a sector contains more cases than expected with respect to the base model, it is said to constitute a *CFA type*. When it contains fewer cases than expected, it is said to constitute a *CFA antitype*.

In most applications, the base model can be expressed in the form of a log-linear model of the form log $\hat{m}$ = 𝑋𝜆, where $\hat{m}$ is the base model-estimated cell frequency, *X* is the design matrix that contains all effects that are not of interest, and *λ* is the vector of model parameters. Tests concerning the cell-specific residuals of this model, that is, 𝜖 = 𝑚 − $\hat{m}$, are used to determine whether a cell constitutes a CFA type or antitype. A number of tests have been proposed to make this decision. These tests take into account the sampling scheme, the size of cells, characteristics of the base model, and distributional assumptions (for more detail, see von Eye, & Wiedermann, 2021, 2024 a). CFA tests are performed under protection of the nominal significance threshold *α*.

From the present perspective, it is important to realize that, thus far, the cells of virtually all cross-classifications that are analyzed with CFA contain individuals. That is, a cell of such a cross-classification contains those individuals that exhibit the profile that is characterized by categories of the variables that span the cross-classification. Exceptions to this rule can be found in the few approaches that have been proposed for CFA of individuals (see von Eye, & Wiedermann, 2021; cf., von Eye et al., 2023). In these exceptions, cells contain numbers of events.

In the present article, we argue that, when numbers of events are the unit of analysis, individuals can be used as the ‘variable’ that spans a cross-classification. The resulting table is, then, of the form Variable 1 × Variable 2 × … × Individuals. The number of categories of the Individuals variable is given by the size of the sample of individuals in the study.

The questions that can be asked based on such a crossclassification are standard CFA questions. To give two examples, one can ask

1. whether the variables that span the cross-classification (including the *Individuals* variable, which is represented by *N* – 1 dummies or effects) show any form of relation that results in types and antitypes; the base model that is used to answer this question is that of global first order CFA, that is, a log-linear main effect model; and
2. whether the relations among the *Variable* 1 × *Variable* 2 × etc. that are part of the cross-classification are individual-specific; the base model that is used to answer this question is that of a regional multigroup CFA.

In the first base model, the design matrix contains only the main effects of all variables. The base model is, thus,


$$\log\;\hat{m}=\lambda +\lambda^{V1}+\lambda^{V2}+\ldots +\lambda^{Individuals},$$

where the variables that span the cross-classification are given in the superscripts. When this model is rejected, interactions are bound to exist. When the Individuals variable is considered a moderator, this hypothesis is not represented in this model.

To test a moderator hypothesis, we need a different model. The base model for the second example contains the main effects of all variables and all possible interactions among the substantive variables, that is, *V*1, *V*2, etc. This model can be expressed as


$$\log\;\hat{m}=\lambda +\lambda^{V1}+\lambda^{V2}+\ldots +\lambda^{Individuals}+\lambda^{V1,V2}+\ldots$$

When there are more than two variables in addition to the Individuals variable, second and higher order interactions among the substantive variables are also included in the model. When this model is rejected, interactions are bound to exist that relate the Individuals variable with the substantive variables. Expressed differently, when this model is rejected, the interactions among the substantive variables differ across the individuals. The Individuals variable functions in this case as moderator. Types and antitypes reflect individual-specific local deviations between, on the one hand, the model expectations that were estimated under the assumption that the interactions among the substantive variables are unchanged across individuals and, on the other hand, observed event frequencies.

The model proposed here distinguishes between two groups of variables. The first group contains the moderator, and the second group contains the substantive variables. As such, the model is a special case of the two-groups-of-variables models proposed by von Eye and Wiedermann (2024) and has the form


$$\log\;\hat{m}=\lambda_{M}+\wedge_{v}[V_{1},\ldots ,V_{j}],$$

where *M* indicates the moderator and the *V* indicates the substantive variables. 𝜆_𝑀_ is the parameter that is estimated for the moderator, and *Λ_V_* is the vector of the parameters that are estimated for the *j* substantive variables and all their interactions. In terms of design matrices, this model can equivalently be expressed as


$$\log\;\hat{m}=[X_{M}|X_{V}][\begin{array}{c} \lambda_{M}\\ \wedge_{V} \end{array}],$$

where *X_M_* is the part of the design matrix that specifies all effects of the moderator(s), and *X_V_* is the part of the design matrix that specifies all effects of the *V* variables. At this point, there is no provision for interactions that link the moderator and the *V* variables. In the following section, we present a real-world data example.

## Stress and Beer Consumption in Alcoholics

For the following example, we use data from a longitudinal study on the development of alcoholism (Perrine et al., 1995). A sample of 50 males who had identified themselves as alcoholics indicated daily the quantity of alcohol they had consumed the day before, and the stress they had experienced on the day of alcohol consumption (for earlier analyses of these data, see, e.g., von Eye, & Wiedermann, 2021, 2024 a,b; Wiedermann & von Eye, 2016). Alcohol consumption was coded as number of beers consumed on a day. Stress was coded on a scale from 0 to 10, with 0 indicating no stress and 10 extreme stress. To keep the tables manageable in size and to reduce sparsity, the two variables were recoded as follows. When a respondent reported having consumed more than 7 beers on a particular day, the number was set to 8. The maximum number of beers that a respondent had reported was 48. This is an extreme outlier, but numbers of beer greater than 7 and less than 48 were rare. Similarly, values of stress greater than 7 were also set to 8.

The first question with which we approach these data concerns the relation between beer consumption and stress. Specifically, we ask whether a relation between these two variables exists on average, that is, disregarding possible individual differences. This question is variable-oriented. The second question is person-oriented. Here, we ask whether the relation between beer consumption and stress varies over the 50 respondents. To answer the second question, we perform a CFA in which ‘Individual’ functions as a moderator.

Table 1 displays the cross-classification of the two variables Stress and Beer consumption (top panel) and the standardized residuals of a Chi-square analysis (bottom panel). A total of 27,305 beers had been consumed over the observation periods that ranged from eight days through 808 days.

Table 1Cross-classification of the association between Stress and Beer consumption

**Table:** 

STRESS (rows) by BEER (columns) (observed frequencies)										
	0	1	2	3	4	5	6	7	8	Total
0	2,238	293	303	306	206	211	174	212	640	4,583
1	1,048	188	207	166	170	130	121	93	316	2,439
2	1,865	283	320	301	270	192	176	148	412	3,967
3	1,562	237	294	251	201	162	167	95	305	3,274
4	1,521	234	261	254	236	146	151	85	261	3,149
5	1,818	306	273	331	320	205	181	74	198	3,706
6	1,413	291	243	271	227	117	101	39	121	2,823
7	952	187	166	203	129	79	89	55	128	1,988
8	584	109	84	171	70	58	84	52	164	1,376
	13,001	2,128	2,151	2,254	1,829	1,300	1,244	853	2,545	27,305

**Table:** 

STRESS (rows) by BEER (columns) (residuals)									
	0	1	2	3	4	5	6	7	8
0	1.196	-3.396	-3.054	-3.718	-5.764	-0.487	-2.408	5.752	10.298
1	-3.325	-0.151	1.072	-2.490	0.518	1.288	0.937	1.925	5.881
2	-0.549	-1.488	0.424	-1.463	0.262	0.228	-0.352	2.162	2.197
3	0.079	-1.137	2.247	-1.172	-1.236	0.490	1.461	-0.720	-0.009
4	0.559	-0.729	0.821	-0.369	1.726	-0.321	0.629	-1.348	-1.897
5	1.272	1.011	-1.109	1.434	4.554	2.150	0.936	-3.882	-7.932
6	1.878	4.786	1.382	2.487	2.756	-1.501	-2.435	-5.238	-8.762
7	0.177	2.576	0.750	3.036	-0.361	-1.609	-0.165	-0.902	-4.209
8	-2.780	0.170	-2.343	5.387	-2.309	-0.928	2.691	1.375	3.157

The overall Pearson Chi-square for Table 1 is 681.275 (*df* = 64; *p* < 0.001). This value suggests that there exists a strong relation between beer consumption and stress. The distribution of the standardized residuals indicates that low stress (Levels 1 and 2) and high beer consumption (Levels 7 and 8) are jointly reported more often than compatible with the assumption of independence, but so is high stress (Level 8+) and high beer consumption (Level 8+). Average to high stress (Levels 5 – 7) and high beer consumption are less often jointly reported than compatible with the assumption of independence.

We now ask whether this pattern (for more detail, see the second panel of Table 1) applies to all or most of the individual respondents. According to Estes (1956; cf. Estes, & Maddox, 2005; cf. von Eye, & Bergman, 2003), there is no ‘average man,’ and it is highly hazardous to generalize from averaged results to the individual. Molenaar (2004), therefore, proposes averaging parameters instead of raw scores. In the present example, Estes’ statement would lead to the hypothesis that the patterns that carry the association between stress and beer consumption vary across individuals.

To test this hypothesis, we perform a CFA in which the ‘variable’ Individual (ID) serves as moderator. The table that we study is the ID × Stress × Beer consumption cross-classification. This table contains, for each respondent, the Stress × Beer consumption cross-classification. It is of size 50 × 9 × 9. Using CFA, there are three options for analysis:

1. CFA of the Stress × Beer consumption cross-classification separately, for each respondent,
2. First order CFA of the entire table; and
3. CFA of the entire table in which ID is treated as moderator.

The CFA base model for the first of the three options would be


$$\forall_{i}\log\;\hat{m_{l}}=\lambda_{i}+\lambda_{i}^{Stress}+\lambda_{i}^{Beer},$$

where *i* indexes the respondents. While doable, this analysis would not formally and statistically treat ID as moderator. Therefore, we do not report the results of this CFA in this context.

The CFA base model for the second of the three options would be


$$\log\;\hat{m}=\lambda +\lambda^{ID}+\lambda^{Stress}+\lambda^{Beer},$$

where the parameters are estimated for the entire cross-classification. Types and antitypes from this base model suggest that there are relations among the three variables. However, while some of these types and antitypes might reflect Stress × Beer consumption associations, ID × Stress associations, or ID × Beer consumption associations, others might reflect ID × Stress × Beer consumption associations. Therefore, some types and antitypes might not speak to the hypothesis that ID functions as moderator, and we do not report the results from this analysis either.

The CFA base model for the third of the options given above is


$$\log\;\hat{m}=\lambda +\lambda^{ID}+\lambda^{Stress}+\lambda^{Beer}+\lambda^{Stress\times Beer}.$$

This model leaves only relations open that link ID with Stress and Beer consumption. These relations suggest that ID is a moderator. This implies that the relation between Stress and Beer Consumption depends on ID, that is, the relation is subject-specific. In the following paragraphs, we discuss the results from this analysis.

The overall LR Chi-square for this base model is 50,415.125 (*df* = 3,920; *p* < 0.001). This extreme result suggests that moderator effects are strong and that subject-specific types and antitypes are bound to exist. This result strongly confirms Estes’ (1956) conjecture according to which there exists no average man: not a single respondent fails to significantly deviate from the base model and, thus, from the model reported in Table 1.

Some of the types and antitypes are extreme indeed. A good number of CFA *z*-scores are greater than *z* = 10. One of these even reaches the value of 64.982^1^ (Cell 1 8 for Respondent 3005). This type suggests that this respondent reported far more often than compatible with the base model that he consumed not a single beer on days of no stress (he reported this for 96 days; expected had been 2.089 days). One of the most extreme antitypes is constituted by Cell 1 1 for Respondent 3035. This respondent reported that he consumed on just one day no beer when he experienced no stress. For this pattern, 66.025 days had been expected (*z* = -8.005; *p* < 0.001).

The number of types and antitypes is very large. Therefore, instead of interpreting every single one of them, we search for patterns. This search is performed visually. The following five patterns of types and antitypes stand out:

1. *No stress - no beer*; this is depicted in Table 2.

**Table. 2:** No Stress – no Beer pattern (A indicates antitype)

Stress	Beer								
	0	1	2	3	4	5	6	7	8+
0		A	A	A	A	A	A	A	A
1		A	A	A	A	A	A	A	A
2+									

Respondents that show this pattern report unexpectedly small numbers of days on which they consume beer when there is no or only little stress. An example of this pattern can be found, for instance, in Respondent 3000.

2. *Strong stress – many beers*; this is depicted in Table 3.

**Table 3:** Strong stress – many beers pattern (T indicates type)

Stress	Beer					
	0 - 3	4+	5	6	7	8+
0 - 6						
7		T	T	T	T	T
8+		T	T	T	T	T

This pattern describes respondents who report unexpectedly many days on which they consume large numbers of beer, in particular when stress is elevated. An example of this pattern can be found, for instance, in Respondent 3004.

*3. No stress – many beers; this is depicted in Table 4*.

**Table 4:** No stress – many beers pattern

Stress	Beer			
	0 - 5	6	7	8+
0		T	T	T
> 0				

This pattern describes respondents who report more days than expected on which they consume six or more beers when there is no stress. Respondent 3005 is an example. This pattern can be viewed as the antithesis of the first pattern.

*4. Elevated stress – elevated number of beers pattern; this is depicted in Table 5*.

**Table 5:** Elevated stress – elevated number of beers

Stress	Beer			
	0 - 2	3	4	5+
0 - 4				
4 - 9		T	T	

This pattern describes respondents who report on unexpectedly large numbers of days that they consumed three or four beers when stress was elevated or high. Respondent 3011 is an example of this pattern.

*5. All or nothing when stress is high; this pattern is depicted in Table 6*.

**Table 6:** All or nothing under high stress pattern

Stress	Beer		
	0	1 - 7	8+
0 - 6			
7+	T		T

**Figure 1:**
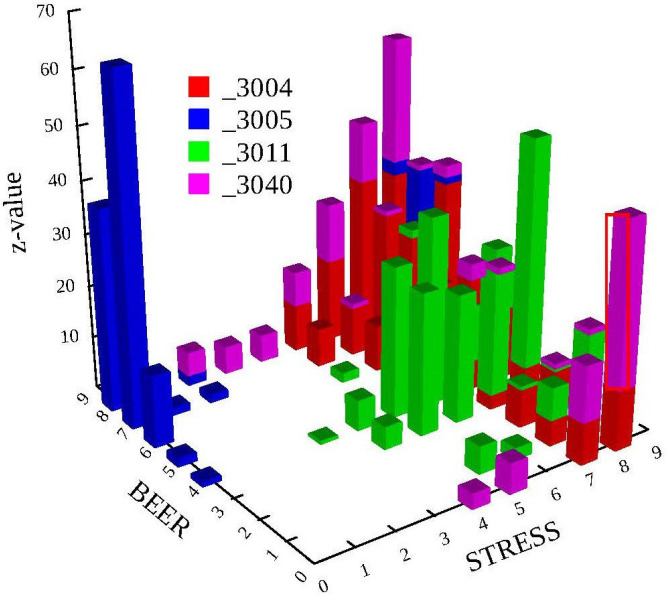
Type patterns of Respondents 3004, 3005, 3011, and 3040

This pattern describes respondents who report surprisingly many days on which they consume either no beer at all or more than eight beers on days with high stress. Respondent 3040 is an example. Figure 1 displays these patterns for the sample cases 3004, 3005, 3011, and 3040.

Respondent 3005 exhibits its strongest types when there is no stress (columns in blue). He thus represents the no stress – many beers pattern. The strongest types exhibited by Respondent 3004 can be found for high stress (columns in red). Types also exist for the high stress – few beers pattern. These types, however, are less extreme than those for the high stress – many beers relation. Respondent 3011 exhibits his strongest types for the elevated stress – elevated number of beers pattern (columns in green). There are no types when stress or beer are low or high. Respondent 3040 responds to high stress in an unpredictable manner. He reports either more days than expected with high beer consumption or little to no beer consumption (columns in purple).

Other patterns do exist. In many respondents, however, the type – antitype pattern does not follow a particular form. Still, even in these respondents, the existence of types and antitypes points at an association between stress and beer consumption. There is no respondent that shows no type or antitype, and the distribution of types and antitypes in each of the respondents differs from the one suggested by Table 1.

To conclude, these results present yet another example of the often-discussed finding that parameters that describe samples may not be generalizable to the individual (e.g., von Eye, & Bergman, 2003). The present data suggest that not a single individual can be properly described based on the sample parameters. Evidently, stress and beer consumption are related, but the characteristics of this relation vary from respondent to respondent.

## CFA to Discriminate Groups of the Moderator Individuals

In the following section, we ask whether an informed classification can result in groups of individuals who differ in variables that were not used in the original moderator analysis. As in the moderator analysis, we use CFA to answer this question. To derive a base model, consider *P*, a variable that represents patterns of variable relations. Each pattern represents a group of individuals with similar patterns. Consider also variables *D_1_, D_2_*, …, *D_j_*, that is the variables used to discriminate among the patterns. In parallel to the question asked in the context of the moderator analysis, we ask here whether the pattern variable represents groups of individuals who differ in the relations among the discrimination variables. The base model, thus, contains,

1. the main effects of all variables in the analysis, and
2. all possible interactions among the discrimination variables.

When this model is rejected, the pattern categories differ in the relations among the discrimination variables. The model is, in parallel to the moderator CFA model,


$$\log\;\hat{m}=[X_{P}|X_{D}][\begin{array}{c} \lambda_{p}\\ \wedge_{D} \end{array}],$$

where *P* indicates the pattern variable (that is, the moderator) and *D* indicates the discrimination variables. In the following section, we continue the example in which we analyze the longitudinal data collected in a sample of alcoholic men.

## Mood and Health in Alcoholics

For the following example, we continue the analysis of data from a longitudinal study on the development of alcoholism (Perrine, et al., 1995). We ask whether the individuals who were analyzed in the above section can be grouped based on their patterns of relations between beer consumption and stress, and whether these groupings differ in their relations between their responses to mood and health-related questions.

To create the pattern variable that represents groups of variable relations, we used the patterns that stood out in the first part of the data example. Specifically, we defined the following four groups (see Figure 1):

1. Unexpectedly high or low beer consumption regardless of stress level. This group subsumes patterns as shown in Tables 2, 3, and 4, as well as patterns in which unexpected levels of beer consumption are constant across all stress levels;
2. Types and antitypes in the corners of the Stress × beer consumption cross-classification (low stress with low as well as high beer consumption and high stress also with low as well as high beer consumption). An example of this pattern is shown in Table 5.
3. Elevated stress in tandem with elevated beer consumption. This pattern shows types predominantly in the center part of the Stress × beer consumption crossclassification (cf. Table 4).
4. Mix of patterns and no clear patterns of types and antitypes.

To distinguish between individuals who exhibit these four patterns of relations, we use the variables self-reported mood and self-reported health. Mood was coded on a scale from 0 to 10, with 0 indicating sour mood and 10 excitation. Health was also coded on a scale from 0 to 10, with 0 indicating subjectively poor health and 10 indicating subjectively perfect health. As in the first part of the data example, to keep the tables manageable in size and to reduce sparsity, the two variables were re-coded to have fewer categories. When a respondent reported experiencing mood at a level below 4, on a particular day, the number was set to 4. The maximum level that a respondent had reported was set to 8. Similarly, values of subjective health were re-coded to range from 4 through 9.

To discriminate the four pattern groups based on their subjective mood and health ratings, a number of variable-oriented methods of analysis can be considered. Here, we employ methods for person-oriented research, specifically, CFA. We attempt to explore whether the relations among subjective mood and health ratings are moderated by the pattern groups. We specified the base model


$$\log\;\hat{m}=\lambda +\lambda^{P}+\lambda^{Mood}+\lambda^{Health}+\lambda^{Mood\times Health}.$$

This model leaves only relations open that link Pattern (*P*) with Mood and Health. These relations suggest that *P* is a moderator. This implies that the relation between Mood and Health depends on the pattern that describe the Mood × Health relation, that is, it is pattern-specific. In the following paragraphs, we discuss results from this analysis.

The overall LR Chi-square for this base model is 4,785.873 (*df* = 87; *p* < 0.001). This extreme value suggests that moderator effects are strong. Specifically, for each pattern category, we find extreme local deviations from the expectancies that were estimated under the above base model. The most extreme CFA type comes with a *z*-score of 24.73, for the mood – health levels 4 4 for the second pattern. 247.74 days with this pattern had been expected, but 637 had been reported. This type suggests that the second pattern differs from the other patterns in particular when the respondents indicate that their mood was sour and their health was poor, on a particular day. The most extreme antitype comes with a *z*-score of 21.465, for the mood – health levels 9 4, also for the second pattern. 441 days were reported with this pattern, but 1,177.578 had been expected. This antitype suggests that it is rare beyond expectation that respondents in Pattern 2 were excited on days during which they rated their health as poor.

In all, the number of types and antitypes in the Pattern × Mood × Health cross-classification is very large. For some patterns, there are less than five cells that do not constitute types or antitypes. We conclude, just as in the first part of the data analysis, that the relations among the psychological variables subjective Stress, Mood, and Health and the amount of beer consumed in self-proclaimed alcoholics are far from universal.

## Discussion

In this article, we discuss methods of analysis for the person-oriented question of how variable relations differ across individuals and groups of individuals. This question is answered with CFA. The base model is specified such that types and antitypes can emerge only when an interaction exists such that variable relations are specific to the individual or the group of individuals.

As an alternative, we discussed performing CFAs separately for each individual and, then, comparing type and antitype patterns. While doable, this alternative suffers from the lack of a statistical basis for the comparison of type and antitype patterns (CFA for the comparison of multiple groups has yet to be fully developed). In addition, the estimation of expected cell frequency uses, in each case, only information from the individual, that is, individual-specific uni- or multivariate marginal probabilities. In contrast, the method proposed here uses marginal probabilities from the entire sample.

This difference is illustrated here using Respondent 3004. Table 7 presents the observed cell frequencies for this respondent. Table 8 shows the standardized residuals that are estimated for the model of independence of Stress from Beer Consumption. Table 9 displays the standardized residuals from the model proposed in this article.

**Table 7:** Observed cell frequencies of Respondent 3004, under two models

STRESS	BEER								
	0	1	2	3	4	5	6	7	8+
0	1	0	0	0	0	0	1	0	0
3	1	0	1	0	0	0	1	2	3
4	0	0	0	0	0	0	0	0	4
5	1	0	0	0	3	3	3	12	27
6	7	0	1	1	5	11	17	11	33
7	66	16	20	12	14	28	41	34	61
8+	61	16	15	17	21	23	53	26	69

**Table 8:** Standardized residuals from model of independence between Stress and Beer Consumption – estimated for Respondent 3004

STRESS	BEER								
	0	1	2	3	4	5	6	7	8+
0	1.038	-0.294	-0.316	-0.284	-0.340	-0.419	1.229	-0.479	-0.729
3	-0.393	-0.587	0.952	-0.569	-0.681	-0.837	-0.224	1.132	0.601
4	-0.859	-0.415	-0.447	-0.402	-0.481	-0.592	-0.791	-0.677	2.851
5	-2.675	-1.454	-1.563	-1.408	0.095	-0.624	-1.684	2.696	3.879
6	-2.228	-1.926	-1.588	-1.328	0.007	1.263	0.970	0.366	2.128
7	1.646	0.960	1.425	0.056	-0.710	0.479	-0.688	0.095	-1.877
8+	0.728	0.838	-0.002	1.385	0.852	-0.656	0.866	-1.444	-1.221

**Table 9:** Standardized residuals from model of independence between ID and the Stress × Beer Consumption association – estimated for Respondent 3004 from the entire sample

Stress	Beer								
	0	1	2	3	4	5	6	7	8+
0	-7.670	-2.822	-2.869	-2.884	-2.366	-2.395	-1.715	-2.400	-4.170
1	-5.337	-2.260	-2.372	-2.124	-2.149	-1.880	-1.813	-1.590	-2.930
2	-7.119	-2.773	-2.949	-2.860	-2.709	-2.284	-2.187	-2.005	-3.346
3	-6.362	-2.538	-2.473	-2.612	-2.337	-2.098	-1.661	-0.362	-1.837
4	-6.429	-2.522	-2.663	-2.627	-2.532	-1.992	-2.026	-1.520	-1.161
5	-6.886	-2.884	-2.724	-2.999	-1.932	-1.089	-0.865	7.044	9.320
6	-5.067	-2.812	-2.181	-2.345	-0.471	4.386	8.605	9.656	16.385
7	7.890	4.843	7.293	2.760	5.605	17.645	24.809	26.588	30.842
8+	11.329	7.576	8.417	5.731	13.847	17.065	33.569	20.683	30.574

The comparison of Tables 8 and 9 shows clearly that the type – antitype patterns differ. The overall LR Chi-square for the base model of independence between Stress and Beer consumption of Respondent 3004 is 111.569 (*df* = 48; *p* < 0.001). We can conclude that, for this respondent, there ex-ists an association between beer consumption and subjective stress. Types and antitypes do emerge. For example, when the stress level is elevated (Stress = 5), the respondent reports drinking seven or more beers on significantly more days than expected. Under the same stress level, drinking no beer occurs less often than expected. When, however, information from the entire sample is the basis for the estimation of expected cell frequencies, the dramatic type – antitype pattern shown in the Table 9 emerges.

These differences reflect the differences between the CFA base models that are estimated. The base model for Table 8 is estimated to identify those cells that carry the individual Stress × Beer Consumption relation. The base model for Table 9 is estimated to answer the question whether individual respondents differ in their Stress × Beer Consumption relations.

There are many ways to extend the models proposed here. Here, we point at six options. First, when the number of individuals to be compared is small, two- or three-group CFA can be employed (this was proposed by von Eye & Mun, 2013a). This approach allows one to compare individuals directly in each configuration.

Second, assumptions concerning the variables in a study can be taken into account. For example, when variables are assumed to be normally distributed, or when a table is incomplete, or both, the base model can be adjusted accordingly (von Eye & Wiedermann, 2024a). This applies accordingly when variables are ordinal in nature.

Third, the approach proposed here implied a change in the unit of analysis. The cells do not contain people but events. The moderator variable identified the individuals that experience or report the events. Linking the present approach with standard CFA of people, moderator variables can be specified to identify the moderating effects of units of time, geographical locations, social contexts, or demographic units. When these or similar variables are used as moderators, the cells of the cross-classification under study can contain either events or people.

Fourth, in the discussion and the example in this article, the moderator was just one variable. Multiple moderators are conceivable as well. For instance, development can be investigated when individuals are examined along a series of temporal junctures, in various social contexts with and without temporal ordering, or in the contexts of various activities.

When multiple moderators are used, the approach proposed here can be viewed as a person-oriented variant of CFA of two groups of variables as it was proposed by von Eye and Wiedermann (2024b). In this case, more complex base models can be considered, even those that include higher order interactions among variables from the two variable groups and distributional assumptions.

Fifth, the options listed here can be employed in combination. For example, multiple covariates and multiple moderators can be used in the same model.

Finally, each of the methods proposed and discussed here was presented in an exploratory context. As is well known, however, CFA can also be applied in confirmatory contexts (for examples, see von Eye & Wiedermann, 2021). To give an example, consider the hypothesis that the drinking patterns of the respondents analyzed in the above examples depend on time spent in jail. In jail, drinking alcohol is prohibited. Given the data, one could test the hypotheses that, after release from jail, (1) whereas for some respondents drinking remains at levels below those before jail time, (2) for others, drinking resumes at elevated levels, and that regardless of stress.

It should be noted that the approach proposed in this article is realistic only when the sample at hand is not too large because the interpretation of individual CFA results can be tedious when large numbers of individuals are involved. On the other hand, the amount of events that individuals report must be large in order to enable configural analysis at the level of the individual.

Finally, we note again that the approach proposed here lies in the heart of person-oriented and idiographic research, even more so than standard CFA. In standard CFA, the cells of a cross-classification contain individuals with identical profiles, and the question is answered whether, with respect to the base model, more or fewer individuals are positioned in each cell. Here, the structure of a cross-classification is compared over individuals.

## Data Availability

The data in the tables may freely be used.

## Footnotes

1 It is well known that, in samples as large as this one, even small effects can be highly significant. Using *p*-values to identify types and antitypes can, therefore, be misleading. Here, we use *p*-values nevertheless because we (1) adjust the nominal significance threshold to the number of tests performed, and (2) we focus on extreme discrepancies between observed and expected cell frequencies as well as on patterns of types and antitypes (see below).

## Action editor

Lars-Gunnar Lundh served as action editor for this article.

## Open access

This article is distributed under the terms of the Creative Commons Attribution 4.0 International License (http://creativecommons.org/licenses/by/4.0/), which permits unrestricted use, distribution, and reproduction in any medium, provided you give appropriate credit to the original author(s) and the source, provide a link to the Creative Commons license, and indicate if changes were made.
